# Correlates of HIV detection among breastfeeding postpartum Kenyan women eligible under Option B+

**DOI:** 10.1371/journal.pone.0216252

**Published:** 2019-05-31

**Authors:** Mary Chan, Eric Munene Muriuki, Sandra Emery, Ruth Kanthula, Vrasha Chohan, Lisa M. Frenkel, Anna Wald, Bhavna Chohan, Julie Overbaugh, Alison C. Roxby

**Affiliations:** 1 Department of Epidemiology, University of Washington, Seattle, Washington, United States of America; 2 Institute for Tropical and Infectious Diseases, University of Nairobi, Nairobi, Kenya; 3 Fred Hutchinson Cancer Research Center, Seattle, Washington, United States of America; 4 Seattle Children’s Research Institute, Seattle, Washington, United States of America; 5 Department of Medicine, University of Washington, Seattle, Washington, United States of America; 6 Department of Pediatrics, University of Washington, Seattle, Washington, United States of America; 7 Department of Laboratory Medicine, University of Washington, Seattle, Washington, United States of America; 8 Department of Global Health, University of Washington, Seattle, Washington, United States of America; 9 Kenya Medical Research Institute, Nairobi, Kenya; 10 Division of Human Biology, Fred Hutchinson Cancer Research Center, Seattle, Washington, United States of America; 11 Division of Public Health Sciences, Fred Hutchinson Cancer Research Center, Seattle, Washington, United States of America; University of Pittsburgh Centre for Vaccine Research, UNITED STATES

## Abstract

**Background:**

The Option B+ strategy streamlines delivery of HIV antiretroviral therapy (ART) to pregnant women, but concerns remain about ART treatment adherence and long term outcomes.

**Methods:**

We conducted a retrospective analysis of a cohort of HIV-positive, postpartum breastfeeding women receiving ART via Option B+ in Nairobi, Kenya. The primary outcome was virologic failure in plasma (HIV RNA >1000 copies/mL), and detection in breast milk (>150 copies/mL) and endocervical secretions (>100 copies/mL) at 2 postpartum timepoints. Correlates of virologic failure were assessed using univariate tests and multivariate logistic regression.

**Results:**

Of 42 women at 6–14 weeks postpartum, 21.4% of women had HIV RNA detected in plasma; 14.3% in breast milk, and 23.7% in endocervical secretions. At 18–24 weeks postpartum, the percentages were 21.1%, 7.1%, and 14.3%, respectively. Younger maternal age, intent to breastfeed for longer, and later ART start in pregnancy were significantly associated with plasma virologic failure (p < 0.05 for each). Odds of plasma virologic failure at 6–14 weeks postpartum were 1.25 times higher (95% CI 1.04, 1.51) for each increase in week of gestation at ART initiation. Only 3 women had resistance mutations to their regimen.

**Conclusions:**

Despite months of ART, nearly one-quarter of the women in our cohort did not achieve plasma virologic suppression in the postpartum period. After adjusting for time on ART, earlier ART initiation in pregnancy was significantly associated with plasma suppression. Our findings suggest that postpartum HIV RNA monitoring in Option B+ programs will be needed to achieve elimination of MTCT.

## Introduction

Treatment of pregnant and postpartum women with antiretroviral therapy (ART) can greatly reduce perinatal HIV transmission and improve maternal health by suppressing HIV viral replication, but limitations in treatment initiation, adherence, and retention in care impede its effectiveness [1]. The Option B+ strategy directs HIV-positive pregnant and breastfeeding women to initiate ART regardless of clinical status or CD4+ cell count, and continue therapy for life [2]. This strategy eliminates treatment delays imposed by CD4+ testing and simplifies the HIV treatment algorithm, facilitating ART initiation and continuation. By targeting pregnant and breastfeeding women, Option B+ addresses maternal disease progression and mother-to-child transmission (MTCT) of HIV in current and future pregnancies, as well as sexual transmission of HIV [3].

Successes associated with Option B+ have included earlier ART initiation in pregnancy, earlier clinical stage of women initiating treatment, increased duration of time that women receive ART before delivery, and decrease in the proportion of young infants (up to 12 weeks of age) becoming infected [2, 4–6]. However, women who initiate ART for pregnancy or breastfeeding are consistently shown to have lower ART adherence and retention rates, particularly in the postpartum period, than women who initiate therapy for clinical stage or CD4+ count [5, 7–9]. In the postpartum period, insufficient viral suppression places infants at risk for infection through breastfeeding, and could result in as many new pediatric HIV infections as are acquired among fetuses and newborns during pregnancy through delivery [10]. Factors leading to incomplete suppression of HIV specific to postpartum mothers on Option B+ may include younger age, fears about a lifetime commitment to ART, and social stigma [11–13]. Less is known about how drug resistance is currently affecting success of Option B+, since many women have already received short-course ART regimens in prior pregnancies. Studying HIV in different biologic compartments is also important to understanding risk of transmission from postpartum women. Endocervical HIV increases risk of HIV during delivery, as well as increasing risk to sexual partners of postpartum women, and breast milk HIV increases risk from breastfeeding. While these compartments typically have much lower levels of HIV detected than contemporaneous plasma measurements, as transmission sites they remain important to measure.

In 2014, Kenya began scaling up Option B+ rollout, and by October 2015, Option B+ had been adopted by over 90% of all facilities offering programs on maternal or infant health [14]. Between 2009 and 2015, Kenya’s MTCT rate at six weeks following birth declined from 8% to 5%, and its MTCT transmission rates, including the breastfeeding period, dropped from 21% to 8% [10, 15]. However, gaps in coverage remain: in a large study conducted between 2014–2015, 6.9% of Kenya’s infants tested positive for HIV [16], and in 2017, UNICEF estimated that 8000 Kenyan children ages 0–4 had incident HIV [10, 17]. This study examines the distribution of virologic failure in plasma, breast milk, and endocervical secretions among postpartum breastfeeding mothers using ART while on Option B+ in Kenya, and explores sociodemographic characteristics that could be associated with virologic failure.

## Methods

### Study design and population

This prospective cohort study enrolled postpartum women engaging in family planning at a public urban primary care clinic in Nairobi, Kenya. Ethical approval for the parent study was granted by the University of Washington Institutional Review Board and the Ethical Review Committee of Kenyatta National Hospital; all participants provided written informed consent.

HIV-positive women who were not pregnant, between 6 weeks and 3 months postpartum, breastfeeding, not using family planning, choosing to start contraception, and asymptomatic for illness were recruited. Midway through recruitment, Option B+ was commenced and ART was offered to HIV-positive women if they were breastfeeding. For this analysis, we included all HIV-positive women on ART who enrolled at least 3 full months after the date of Option B+ rollout to ensure that they had the opportunity to receive Option B+ for sufficient time to achieve viral suppression [18].

### Data collection

Questionnaires were verbally administered to postpartum women at enrollment (6–14 weeks postpartum), and during study follow-up visits (18–24 weeks postpartum), including sociodemographic, clinical, and treatment information. Plasma, endocervical swabs, and breast milk were collected at each visit for HIV-1 RNA, measured by a prototype HIV-1 viral RNA assay (Gen-Probe/Hologic, San Diego, CA) validated for each specimen type [19].

Participants were grouped into those with virologic failure and those with virologic suppression based on the following HIV RNA thresholds: for plasma, >1000 copies/mL; for breast milk >150 copies/mL, and for endocervical secretions, >100 copies/mL. For both breast milk and endocervical secretions, the threshold was also the lower limit of RNA detection of the assay. For subjects with >1000 copies/mL of HIV RNA in plasma at any visit, plasma was evaluated for HIV genotypic resistance via the oligonucleotide ligation assay (OLA), using probes optimized for subtypes A, D and C prevalent in Kenya [20, 21].

### Statistical methods

Data was entered into RedCap and analyses were performed using STATA/SE 14.2. Our primary outcome was plasma virologic failure. Participant characteristics at 6–14 weeks postpartum were evaluated using descriptive statistics. Participants with plasma virologic failure were compared against those with virologic suppression at the same time point to elicit sociodemographic and clinical correlates of virologic failure. Comparisons were made for both enrollment (6–14 week) and follow-up (18–24 week) timepoints, as women’s adherence to ART may differ in the early and late postpartum periods. Univariate analyses were performed using Fisher’s exact test for categorical variables and the Mann Whitney U test for continuous variables; two-sided p-values were calculated. Cohen’s Kappa was calculated to examine concurrency in virologic failure among multiple compartments [22]. Missing values for variables with over 10% of data missing were treated as additional categories in analyses. Viral load measurements below the limit of detection were recoded to midway between zero and the assay’s lower limit of detection, which was 100 copies/mL for plasma and endocervical secretions, and 150 copies/mL for breast milk [23]. For women for whom weeks of gestation at ART initiation were missing, this value was calculated using infant’s birth date. Weeks of gestation under 0 were recoded as 0 and over 45 were recoded as 45.

Variables determined through univariate analysis to be associated with plasma virologic failure at p-values of 0.20 and under, and biologically plausible for association, were considered for multivariate models constructed using both stepwise and backwards elimination methods. Separate analyses were performed for the 6–14 week and 18–24 week postpartum periods using logistic regression and a 2-sided significance threshold of 0.05.

The clinic started Option B+ in August 2014. We excluded subjects who enrolled before Dec. 1, 2014 to ensure that all subjects were offered ART under Option B+. We did not have access to infant HIV test results, although all infants in the study were tested for HIV at age 6 weeks. HIV viral load testing was technically offered to mothers at the clinic, but in practice, very few women were tested, due to a variety of technical barriers.

## Results

### Characteristics of the study population

Our study recruited 66 HIV positive women, of whom 59 reported being on ART at either enrollment or follow-up. Of these women, 42 enrolled on or after Dec. 1, 2014 (Table 1).The median number of weeks postpartum was 6.9 weeks (IQR 6.6, 10.9). The mean age was 26.9 (± 4.6) years, most were married (n = 35, 83.3%), and 35 (83.3%) had disclosed their HIV status to their partners. At enrollment, all were breastfeeding and intended to breastfeed for 11 (±3.1) months. Nearly all (n = 40, 95.2%) reported being counseled on the importance of ART adherence and all reported being on ART at enrollment. Most had initiated ART during pregnancy (n = 26, 66.7%) at a median of 21.7 (IQR 4.0, 28.0) weeks of gestation while 10, (23.8%), had initiated ART prior to pregnancy. All women were on first-line ART regimens including reverse transcriptase inhibitors, 2 nucleoside analogues paired with non-nucleosoide analogues efavirenz (85% of women) or nevirapine (15% of women); no women were taking protease inhibitors. Thirty-four (81.0%) self-reported good adherence during pregnancy and most reported good adherence during breastfeeding (n = 41, 97.6%), defined as missing at most two doses a month. At 18–24 weeks postpartum, one women reported no longer taking ART, and 38 (90.5%) were still breastfeeding.

**Table 1:** Sociodemographic and clinical characteristics of the study population.

Maternal Sociodemographic and Clinical Characteristics	N (%)*, Median (IQR), or Mean (SD) (N = 42)
**6–14 weeks postpartum**	
Maternal age (years)	26.9 (±4.6)
Married^∞^	35 (83.3%)
Number of previous pregnancies	2.5 (±1.4)
Attended at least some secondary school	18 (42.9%)
Estimated monthly rent (USD°)^i^	42 (±41)
Weeks postpartum at enrollment	6.9 (6.6, 10.9)
Received DMPA contraception	29 (69.1%)
Infant’s father is HIV positive^†^	17 (40.5%)
Partner aware of woman’s HIV status	35 (83.3%)
Infant tested for HIV at ~6 weeks of age^i^	39 (95.1%)
Enrolled in Comprehensive Care Clinic (CCC)	42 (100%)
Months intending to breastfeed	11.3 (±3.1)
Breastfeeding	42 (100.0%)
Counseled about importance of ART adherence	40 (95.2%)
Took ZDV or ART in a previous pregnancy	21 (50.0%)
On ART	42 (100%)
Months between HIV diagnosis and ART start^‡^	
≤ 1 month (30 days)	6 (14.3%)
> 1 month (31 days+)	22 (52.4%)
Timing of ART initiation	
prior to pregnancy	10 (23.8%)
during pregnancy through delivery	28 (66.7%)
upon or after delivery	4 (9.5%)
Gestation (weeks) at ART start	21.7 (4.0, 28.0)
ART adherence during pregnancy^i^	
missed ≤ two doses per month	34 (81.0%)
missed > two doses per month	3 (7.1%)
not on ART^α^	4 (9.5%)
ART adherence during breastfeeding^i^ ^β^	
missed ≤ doses per month	41 (97.6%)
missed > two doses per month	-
not on ART	-
Length of time on ART at 6–14 weeks postpartum, in weeks^iv^	25.5 (17.7, 42.9)
Self-reported CD4 count at ART initiation^iii^	
> 350 cells/mm^3^	7 (16.7%)
≤ 350 cells/mm^3^	7 (16.7%)
Unknown	25 (59.5%)
Lab-reported CD4 count^i^	
> 350 cells/mm^3^	32 (76.2%)
≤ 350 cells/mm^3^	9 (21.4%)
Detectable plasma HIV RNA	12 (28.6%)
Virologic failure detected	
in plasma (>1000 copies/mL)	9 (21.4%)
in breast milk^i^ (>150 copies/mL)	6 (14.3%)
in endocervical swab (>100 copies/mL)	10 (23.8%)
**18–24 weeks postpartum**	
Time elapsed since enrollment, in weeks^ii^	13.0 (12.0, 13.1)
On ART^ii^	39 (92.9%)
Breastfeeding^ii^	38 (90.5%)
Detectable plasma HIV RNA^iv^	12 (28.6%)
Virologic failure detected	
in plasma^iv^ (>1000 copies/mL)	8 (21.1%)
in breast milk^iv^ (>150 copies/mL)	3 (7.1%)
in endocervical swab ^ii^ (>100 copies/mL)	6 (14.3%)

IQR = Interquartile Range; SD = Standard Deviation; USD = US dollars; DMPA = depot-medroxyprogesterone acetate; ART = antiretroviral therapy; ZDV = zidovudine.

* May not sum to 100% due to missing values.

^∞^ Versus single/divorced/separated/widowed.

° Using an exchange rate of 92 USD to 1 Kenyan Shilling.

^I^ 1 missing.

^ii^ 2 missing.

^iii^ 3 missing.

^iv^ 4 missing.

^†^ 13 missing.

^‡^ 14 missing.

^α^ 1 started ART at delivery, and 3 sometime between delivery and enrollment.

^β^ From the time of ART initiation (before enrollment for all women).

### Distribution of virologic failure

Plasma HIV RNA levels above the detectable limit (100 copies/mL) were found in 12 subjects (28.6%) at enrollment; median HIV RNA level was 14096 copies/mL (IQR 1328, 56046). Nine of these subjects (21.4% overall) had plasma virologic failure (> 1000 copies/mL). Of these, 1 subject was able to suppress plasma HIV to ≤ 1000 copies/mL by follow-up, and a different subject, who was initially suppressed, demonstrated plasma virologic failure at the later time point (Table 2). Five of these ten women were found to have resistance mutations, although only 3 women had reverse transcriptase (RT) mutations likely to interfere with their stated ART regimen.

**Table 2:** Subjects with detectable HIV RNA level ^Δ^ at 6–14 weeks or 18–24 weeks postpartum.

Subject	6–14 weeks postpartum			18–24 week postpartum			Resistance mutations*
	plasma	breast milk	endocervical secretions	plasma	breast milk	endocervical secretions	plasma
1	11706	*75*	9165	134	*75*	*50*	No mutations
2	*50*	*75*	*50*	400	*75*	*50*	Not tested
3	34744	2860	190	41910	220	498	K103N (RT)
4	47748	*75*	105	42168	*75*	*50*	No mutations
5	150	*75*	*50*	*50*	*75*	*50*	Not tested
6	16486	*75*	452	24488	*75*	703	M184V, Y181C, H221Y (RT)
7	1836	*75*	122	63802	*75*	6530	No mutations
8	820	*75*	435	1746	*75*	*50*	K65R, M184V, K103N, Y181C (RT)
9	64344	*75*	5865	*(missing)*	*(missing)*	*(missing)*	L90M (PI)
10	*50*	*75*	*50*	*50*	750	*50*	Not tested
11	*50*	*75*	*50*	108	*75*	*50*	Not tested
12	4178	2615	143	5944	*75*	9765	No mutations
13	*50*	*75*	*50*	236	*75*	*50*	Not tested
14	222630	6965	9178	118438	690	6095	M46L (PI)
15	*50*	180	*50*	*(missing)*	*(missing)*	*50*	Not tested
16	83116	340	7305	18278	*75*	1375	No mutations
17	690	520	*50*	*50*	*75*	*50*	Not tested
Total N with virologic failure	9	6	10	8	3	6	
Median HIV RNA*, IQR	14096 (1328, 56046)	1568 (340, 2860)	444 (143, 7305)	12111 (318, 42039)	690 (220, 750)	3735 (703, 6530)	

IQR = Interquartile range; RT = reverse transcriptase mutation. PI = Protease inhibitor mutation.

^Δ^ Values given in copies/mL. Thresholds for virologic failure: plasma >1000 copies/mL, breast milk >150 copies/mL, endocervical secretions >100 copies/mL. Values below the lower limit of detection are in italics.

* Median HIV RNA calculated using only values above the threshold of detection (100 copies/mL for all specimen types).

Note: Plasma testing for HIV mutations conferring drug resistance was performed for all subjects with plasma virologic failure (VL >1000 copies.ml) at either time point.

Among the 6 subjects (14.3%) with breast milk detection at 6–14 weeks postpartum, the median HIV RNA level was 1568 copies/mL (IQR 340, 2860). Three subjects were able to suppress breast milk HIV levels at follow-up and 1 other initially suppressed at enrollment was no longer suppressed at the later time point. Among the 10 women (23.8% overall) with endocervical detection at 6–14 weeks postpartum, 3 became virologically suppressed; no additional subjects had endocervical detection at follow-up. The median HIV RNA level among detectable endocervical secretion specimens was 444 copies/mL (IQR 143, 7305) at enrollment.

RNA detection in plasma showed fair agreement with HIV detection in breast milk and good agreement with HIV detection in endocervical secretions. At 6–14 weeks postpartum, all 9 subjects with plasma virologic failure also had HIV detected in breast milk, endocervical compartments, or both. Percent agreement beyond chance in virologic status was moderate (Cohen’s κ = 0.43) for plasma and breast milk, and almost perfect (Cohen’s κ = 0.93) for plasma and endocervical secretions. Percent agreement beyond chance in virologic status was also notable at 18–24 weeks postpartum; this time poor (Cohen’s κ = 0.28) for plasma and breast milk, and excellent (Cohen’s κ = 0.83) for plasma and endocervical secretions.

### Sociodemographic characteristics associated with plasma virologic failure

Women with virologic failure in plasma at 6–14 weeks postpartum were significantly younger than women who were suppressed, the former having a mean of 24.2 (±4.4) years of age compared to 27.6 (±4.4) years among the latter (p = 0.05). Additionally, women with plasma virologic failure initiated ART at a later time in pregnancy, at 27.0 weeks (IQR 26.0, 32.0), compared to 20.0 weeks’ (IQR 0, 25.0) gestation among suppressed women (p = 0.02). Women with plasma virologic failure trended toward a shorter period of time on ART: 17.3 weeks (IQR 12.1, 30.0) compared to 27.7 weeks (IQR 19.9, 43.9) among those who were suppressed (p = 0.08). No significant differences were detected in CD4+ count between women with failure and suppression at enrollment (Table 3).

**Table 3:** Correlates of postpartum virologic failure.

Maternal Sociodemographic and Clinical Characteristics	6–14 weeks postpartum			18–24 weeks postpartum		
	N (%)*, Median (IQR), or Mean (SD)		p-value	N (%)*, Median (IQR), or Mean (SD)		p-value
	>1000 copies/mL (failure) (n = 9)	≤1000 copies/mL (suppression) (n = 33)		>1000 copies/mL (failure) (n = 8)	≤1000 copies/mL (suppression) (n = 30)	
Maternal age (years)	24.2 (±4.4)	27.6(±4.4)	**0.05**	25.9 (±4.7)	27.1 (±4.2)	0.43
Married	8 (88.9%)	27 (81.8%)	1.00	7 (87.5%)	26 (86.7%)	1.00
Number of past pregnancies	2.3 (±1.2)	2.5 (±1.5)	0.97	2.8 (±1.3)	2.3 (±1.3)	0.23
Attended at least some secondary school	5 (55.6%)	13 (39.4%)	0.46	3 (37.5%)	13 (43.3%)	1.00
Estimated monthly rent (USD°)	66 (±82)	36 (±15)	0.67^i^	67 (±88)	37 (±16)	0.55^i^
Weeks postpartum at enrollment	7.0 (6.7, 7.7)	6.7 (6.6, 10.9)	0.77	6.9 (6.6, 7.4)	6.9 (6.6, 11)	0.58
Received DMPA contraception	7 (77.8%)	22 (66.7%)	0.70	6 (75.0%)	21 (70.0%)	1.00
Disclosed HIV status to partner	8 (88.9%)	27 (81.8%)	1.00	7 (87.5%)	26 (86.7%)	1.00
Months intending to breastfeed	13.3 (±4.0)	10.7 (±2.6)	**0.05**	13.5 (±4.2)	10.8 (±2.6)	0.06
Took ART in a previous pregnancy	4 (44.4%)	17 (51.5%)	1.00	3 (37.5%)	17 (56.7%)	0.44
Timing of ART initiation			0.29			0.11
prior to pregnancy	1 (11.1%)	9 (27.3%)		2 (25.0%)	6 (20.0%)	
pregnancy	6 (66.7%)	22 (66.7%)		4 (50.0%)	23 (76.7%)	
after delivery	2 (22.2%)	2 (6.1%)		2 (25.0%)	1 (3.3%)	
Gestation (weeks) at ART start	27 (26, 32)	20 (0, 25)	**0.02**^i^	26 (12, 36)	20 (8, 27)	0.20
ART adherence			0.21^i^			0.22^i^
missed ≤ 2 doses/month	6 (66.7%)	28 (84.9%)		6 (75.0%)	25 (83.3%)	
missed > 2 doses/month	1 (11.1%)	2 (6.1%)		2 (25.0%)	3 (10.0%)	
not on ART	2 (22.2%)	2 (6.1%)		0 (0%)	1 (3.3%)	
Length of time on ART at 6–14 weeks	17.3 (12.1, 30.0)	27.7 (19.9, 43.9)	0.08^iii^	26.0 (9.9, 66.7)	24.3 (18.9, 39.3)	0.77^ii^
CD4 count 6–14 weeks postpartum			0.38^i^			0.05^i^
> 350 cells/mm^3^	6 (66.7%)	26 (78.8%)		4 (50.0%)	25 (83.3%)	
≤ 350 cells/mm^3^	3 (33.3%)	6 (18.2%)		4 (50.0%)	4 (13.3%)	

IQR = Interquartile Range; SD = Standard Deviation; ART = antiretroviral therapy; DMPA = depot-medroxyprogesterone acetate;

* May not sum to 100% due to missing values.

^i^ 1 missing.

^ii^ 3 missing.

^iii^ 4 missing.

° Exchange rate of 92 USD to 1 Kenyan Shilling.

We did not detect differences between women with plasma virologic failure and suppression in marital status, number of past pregnancies, secondary school education, income, and use of injectable contraception. The groups also did not differ in having partners who were aware of their HIV status, ART adherence during breastfeeding and during pregnancy, or receiving ART in a previous pregnancy. No subjects self-reported missing more than two doses of ART a month during breastfeeding, so this variable was excluded from further analysis.

Maternal age and gestation at ART initiation were not significantly associated with plasma virologic failure at 18–24 weeks postpartum. However, women with plasma virologic failure in late postpartum were significantly more likely to have a CD4+ count of ≤ 350 cells/mm^3^ compared to suppressed women (p = 0.05).

Variables considered for multivariate models included maternal age, gestation at ART initiation, length of time on ART at enrollment (6–14 weeks postpartum), and low CD4+ count at enrollment (Table 4). For each additional week that a pregnancy progressed before ART initiation, the odds of having plasma virologic failure at 6–14 weeks postpartum were 1.25 times higher (95% CI 1.04, 1.51) when adjusted for length of time on ART and low CD4+ count at enrollment. At 18–24 weeks postpartum, the odds of having plasma virologic failure were 1.16 times higher (95% CI 1.02, 1.33) for each later week in pregnancy before ART initiation, adjusted for the same variables.

**Table 4:** Odds ratios of virologic failure in plasma^Δ^.

Maternal Sociodemographic and Clinical Characteristics	6–14 weeks postpartum			18–24 weeks postpartum		
	N	Multivariate aOR (95% CI)	p-value	N	Multivariate aOR (95% CI)	p-value
Maternal age (years)	37	*		34	*	
Gestation at ART start (weeks)		1.25 (1.04, 1.51)	**0.02**		1.16 (1.02, 1.33)	**0.03**
Length of time on ART at enrollment		1.03 (1.00, 1.06)	**0.04**		1.03 (1.00, 1.05)	**0.02**
Low CD4 count at 6–14 wks postpartum		3.74 (0.37, 38.0)	0.27		24.54 (1.54, 391.02)	**0.02**

N = number of women OR = Odds Ratio. aOR = adjusted Odds Ratio. All listed variables were considered for the multivariate model.

^Δ^ Threshold for virologic failure in plasma: >1000 copies/mL.

* Excluded from the final multivariate model.

### Performance of Option B+ cascade

As a measure of health center performance, we assessed women’s exposure to the steps of the PMTCT care cascade. More than 95% of women in the study reported completing all the steps of the care cascade, including taking maternal cotrimoxazole, enrolling in the HIV clinic, starting ART, giving ART prophylaxis to their infant, receiving adherence counseling, exclusively breastfeeding, and testing their infant for HIV at 6 weeks postpartum.

## Discussion

Despite access to Option B+, we found that, both in early and late postpartum, 21% of women had virologic failure in plasma, and those women also were likely to have HIV detected in concurrent breast milk or endocervical secretions, which could impact vertical and sexual transmission in the postpartum period. The goal of Option B+ was to promote earlier initiation of ART during pregnancy, and our data show that the benefits of starting ART earlier in pregnancy extend into the postpartum period. We demonstrate better suppression among women who started ART earlier in pregnancy, even after controlling for length of time on ART. This finding adds to ongoing debate concerning how pregnancy interacts with ART. Women on ART have been shown to have a higher risk of plasma virologic failure if they become pregnant during therapy [24]. Myer *et al*. demonstrated that, among women with pre-ART viral loads of > 4.0 log copies/mL, the probability of achieving a plasma RNA level of ≤ 50 copies/mL by delivery is greater for women initiating ART earlier in gestation [18]. Alternatively, other mechanisms that involve ART administration and adherence could also influence viral suppression in the antepartum and postpartum periods [24].

Our results are consistent with other descriptions of virologic failure and suppression among postpartum women [12, 18]. In a cohort of 150 Ugandan women who were offered Option B+, 80.7% were suppressed in plasma, but follow-up was performed 3–5 years after initiation, and the cohort was ART-naïve upon ART initiation at 12–28 weeks of gestation [4]. Among 434 women in Kenya who initiated ART regardless of CD4+ cell counts, 80% and 79% had undetectable plasma HIV viral loads at 14 and 24 weeks postpartum, respectively, but the cohort had initiated ART at 34 weeks gestation, and nelfinavir or nevirapine-based triple regimens were administered [25]. Our cohort included women on more efficacious and better tolerated single-tablet efavirenz-based regimens, but did not show better virologic outcomes.

Our study also measures HIV load in breast milk and endocervical secretions, to address the actual sites of HIV transmission to infants and sexual partners. Although virologic status in breast milk correlated strongly with plasma, breast milk HIV RNA levels often fluctuate in ART-treated women [23], and a recommended target threshold for virologic suppression in breast milk has not yet been established. Davis et al. found transmission risk to be 3.8 times higher among women with detectable (>40 copies/mL) compared to non-detectable levels of HIV RNA in breast milk, but all transmitting mothers also had at least one postpartum plasma HIV RNA >3500 copies/mL [26]. Detection of HIV in endocervical secretions also matched well against that in plasma. However, variability in endocervical HIV RNA is typically higher than in plasma, and the magnitude of viral shedding in endocervical secretions can remain at transmissible levels even when plasma HIV levels are undetectable [27, 28], although risk of HIV transmission is very low among women with genital HIV RNA levels below 240 copies/mL [29].

About half of the women in our study reported using ART to prevent MTCT in a prior pregnancy. There is documentation that short-course regimens taken in pregnancy result in archived drug resistance in mothers [30], and we were concerned that drug resistance might be a reason for plasma HIV detection. We found 3 women with extensive resistance to RT therapy and 7 women with no RT mutations. This confirmed that adherence was the most probable reason for failure for most women. But the 30% of women with extensive resistance would be unlikely to succeed on their current regimen, even if taking regularly. This highlights the need for both viral load monitoring and resistance testing to achieve elimination of MTCT. The OLA assay used here is a low-cost technology that could be deployed in resource-limited settings.

Presumed poor adherence to treatment among women attending clinic regularly was an unexpected finding of our study. Most women were prescribed single-tablet efavirenz-based regimens, which reliably suppress HIV when taken and which are relatively forgiving if missed occasionally. No subjects in our cohort reported any stock-outs or problems obtaining ART. Drug resistance as discussed above could only explain one-third of treatment failures. ART adherence self-reported by our subjects, particularly while breastfeeding, was substantially higher than typically reported [31]. However, we did find that younger maternal age, a possible barrier to adherence, was significantly associated with early postpartum plasma virologic failure. Younger women have been consistently demonstrated to be more likely than older women to be lost to follow-up [32–34]. Interventions that could improve adherence when combined with enhanced standard of care include text messaging and phone-based interventions, interventions which may resonate with younger women [35, 36].

Other study limitations include our small sample size, which reduced our ability to link other sociodemographic characteristics to poor viral suppression. Our study population was drawn from an ART clinic in the Nairobi area that was exceptionally high-performing, and participants received enhanced support and follow-up for ART adherence, which limits the generalizability of these results to rural, low-performing, or average clinics; or to women not receiving additional ART support as part of a research study. However, we are concerned that our results indicate that more average clinical conditions with minimal extra supports might produce even lower viral suppression than we observed in our study.

Option B+ remains a strategy that shows promise for protecting pregnant woman and their infants in the pregnancy and early breastfeeding period. However, our results were concerning: among a group of women who reported good ART adherence, faced no drug stock-outs, and who attended a high-performing health center regularly, we nevertheless detected more than 1000 copies/ml of HIV in the blood of one in five postpartum women. To date, in most settings, Option B+ programs in Kenya have limited access to HIV viral load testing to assess viral suppression. Our data indicate that even in high-performing clinics, HIV viral load testing could identify women who would benefit from adherence counseling and ART resistance testing, infants who would benefit from ongoing ART prophylaxis during the breastfeeding period, and HIV-negative male partners who could benefit from pre-exposure prophylaxis to prevent sexual transmission. The promise of Option B+ will be incomplete without timely viral load testing for postpartum women.
